# Mutation of *Npr2* Leads to Blurred Tonotopic Organization of Central Auditory Circuits in Mice

**DOI:** 10.1371/journal.pgen.1004823

**Published:** 2014-12-04

**Authors:** Cindy C. Lu, Xiao-Jie Cao, Samantha Wright, Le Ma, Donata Oertel, Lisa V. Goodrich

**Affiliations:** 1 Department of Neurobiology, Harvard Medical School, Boston, Massachusetts, United States of America; 2 Department of Neuroscience, University of Wisconsin School of Medicine and Public Health, Madison, Wisconsin, United States of America; 3 Department of Cell and Neurobiology, Keck School of Medicine, University of Southern California, Los Angeles, California, United States of America; University of Pittsburgh School of Medicine, United States of America

## Abstract

Tonotopy is a fundamental organizational feature of the auditory system. Sounds are encoded by the spatial and temporal patterns of electrical activity in spiral ganglion neurons (SGNs) and are transmitted via tonotopically ordered processes from the cochlea through the eighth nerve to the cochlear nuclei. Upon reaching the brainstem, SGN axons bifurcate in a stereotyped pattern, innervating target neurons in the anteroventral cochlear nucleus (aVCN) with one branch and in the posteroventral and dorsal cochlear nuclei (pVCN and DCN) with the other. Each branch is tonotopically organized, thereby distributing acoustic information systematically along multiple parallel pathways for processing in the brainstem. In mice with a mutation in the receptor guanylyl cyclase Npr2, this spatial organization is disrupted. Peripheral SGN processes appear normal, but central SGN processes fail to bifurcate and are disorganized as they exit the auditory nerve. Within the cochlear nuclei, the tonotopic organization of the SGN terminal arbors is blurred and the aVCN is underinnervated with a reduced convergence of SGN inputs onto target neurons. The tonotopy of circuitry within the cochlear nuclei is also degraded, as revealed by changes in the topographic mapping of tuberculoventral cell projections from DCN to VCN. Nonetheless, *Npr2* mutant SGN axons are able to transmit acoustic information with normal sensitivity and timing, as revealed by auditory brainstem responses and electrophysiological recordings from VCN neurons. Although most features of signal transmission are normal, intermittent failures were observed in responses to trains of shocks, likely due to a failure in action potential conduction at branch points in *Npr2* mutant afferent fibers. Our results show that *Npr2* is necessary for the precise spatial organization typical of central auditory circuits, but that signals are still transmitted with normal timing, and that mutant mice can hear even with these deficits.

## Author Summary

Millions of people suffer from debilitating hearing defects, ranging from a complete inability to detect sound to more subtle changes in how sounds are encoded by the nervous system. Many forms of deafness are due to mutations in genes that impair the development or function of hair cells, which are responsible for changing sound into electrical signals that can be processed by the brain. Both mice and humans carrying these mutations fail standard hearing tests. In contrast, very little is known about the genetic basis of central auditory processing disorders, which are poorly defined and difficult to diagnose, since these patients can still detect sounds. By finding genes that are required for the normal wiring of central auditory circuits in mice, we can investigate how changes at the circuit level affect circuit function and therefore improve our understanding of central auditory processing disorders. Here, we show that the natriuretic peptide receptor Npr2 is required to establish frequency maps in the mouse central auditory system. Surprisingly, despite a dramatic change in circuit organization, *Npr2* mutant mice are still able to respond to sounds with normal sensitivity and timing, underscoring the need for better hearing diagnostic methods in mice as in humans.

## Introduction

The sense of hearing is mediated by precisely organized neural circuits that encode the frequency content, timing, and intensity of sounds. Frequency information is encoded in the spatial organization of hair cells in the cochlea, with high frequencies detected in the base and low frequencies in the apex. SGNs transmit this information to the cochlear nuclei, where their axons bifurcate into an ascending branch that innervates the aVCN and a descending branch that targets the pVCN and DCN. In each of these regions, the systematic innervation by SGN fibers forms frequency maps that maintain the tonotopic order that is established in the cochlea and that is preserved along the auditory pathway. Tonotopy also governs intrinsic connections between neurons in the cochlear nuclei, including tuberculoventral cell projections from the DCN to the VCN [1], [2].

SGN axons are responsible for delivering all acoustic information from the cochlea to the cochlear nuclei. By contacting a variety of target neurons with distinct projection patterns, each SGN feeds information to parallel pathways in the brainstem [3]. Through their ascending branches, SGNs convey auditory signals to bushy cells that are involved in comparing interaural time and intensity for localizing sounds in azimuth [4], [5], [6], [7], as well as to some T stellate cells. Through their descending branches, SGNs innervate T stellate cells that encode the spectrum of sounds [8], [9], [10], octopus cells that mark the onset of sounds [11], [12], [13], and fusiform and giant cells of the DCN that use spectral cues to localize sounds monaurally in the vertical plane [14]. Together, the activation of these diverse populations of cochlear nuclear neurons by SGN axons enables animals to detect, recognize, and locate sounds in their environment.

In order to make the precise pattern of diverse connections that enable the interpretation of sound, developing SGN axons must elaborate a variety of synapses that are tonotopically organized but that show distinct signaling properties depending on the nature of the target neuron. For instance, within one isofrequency lamina of the VCN, SGN axons contact bushy cells, T stellate, and octopus cells and form functionally distinct synapses with each cell type. The branches that innervate bushy cells terminate in unusually large and complex endbulbs of Held that mediate large post-synaptic responses that depress, yet still signal with high temporal precision [15], [16]. In contrast, on T stellate cells, SGN axons form typical bouton endings that induce smaller post-synaptic responses with less depression. Traditionally, the mechanisms that control axon guidance and synaptic function have been studied independently. However, recent evidence indicates that these two events can in fact be linked, as maturation of the calyx of Held does not progress normally in bushy cell axons that fail to cross the midline [17]. Whether the spatial organization of SGN axons is similarly coordinated with subsequent synaptogenesis remains unclear.

One of the primary obstacles towards understanding how functional auditory circuits are assembled is the lack of genetic mutations that disrupt SGN central wiring. As a result, there are no clear predictions for how changes in the pattern of central innervation might impact hearing either in mice or humans. Indeed, although a growing number of genes affecting cochlear function have been implicated in sensorineural deafness in humans [18], almost nothing is known about the genetic basis of central auditory processing disorders, which disrupt central auditory circuit function without obvious loss of hearing sensitivity [19], [20]. Identifying and characterizing genetic mutations that affect the formation of central auditory circuits in mice is an important step towards understanding how these disorders may arise.

During the course of a screen to identify genes required for auditory circuit assembly, we discovered that the natriuretic peptide receptor Npr2 is required for central axon bifurcation in SGNs [21]. Npr2 is a receptor guanylyl cyclase that activates a cGMP-dependent protein kinase signaling cascade upon binding to the C-type natriuretic peptide (CNP), which is expressed dorsally along the length of the embryonic neural tube [22], [23]. In *Npr2* mutant mice, the axons of both dorsal root ganglion (DRG) and cranial ganglion neurons fail to bifurcate [24], [25]. However, interstitial branches can still form from the unbifurcated axons. These observations suggest that Npr2 ensures that bifurcations form in an orderly manner as sensory axons enter the spinal cord and encounter CNP, but that other mechanisms determine how and when axons arborize within their targets [26]. Although Npr2 signaling leads to changes in cytoskeletal-associated proteins [27], [28], [29], how Npr2 affects growth cone behavior is unclear, as CNP can also act as a chemoattractant [28]. In addition, the full extent of Npr2's effects on the organization and function of the mature nervous system has not yet been defined. Since *NPR2* is mutated in human patients with achondroplasia [30], a deeper understanding of the *Npr2* phenotype in an animal model is needed. Here, we seek to gain insight into the mechanisms that govern SGN central wiring by characterizing the long term effects of *Npr2* mutations on both the spatial organization and functional maturation of synaptic connections between SGN axons and their cochlear nuclear targets.

## Results

### SGNs appear normal in the auditory periphery of *Npr2* mutant mice

**Figure 1:**
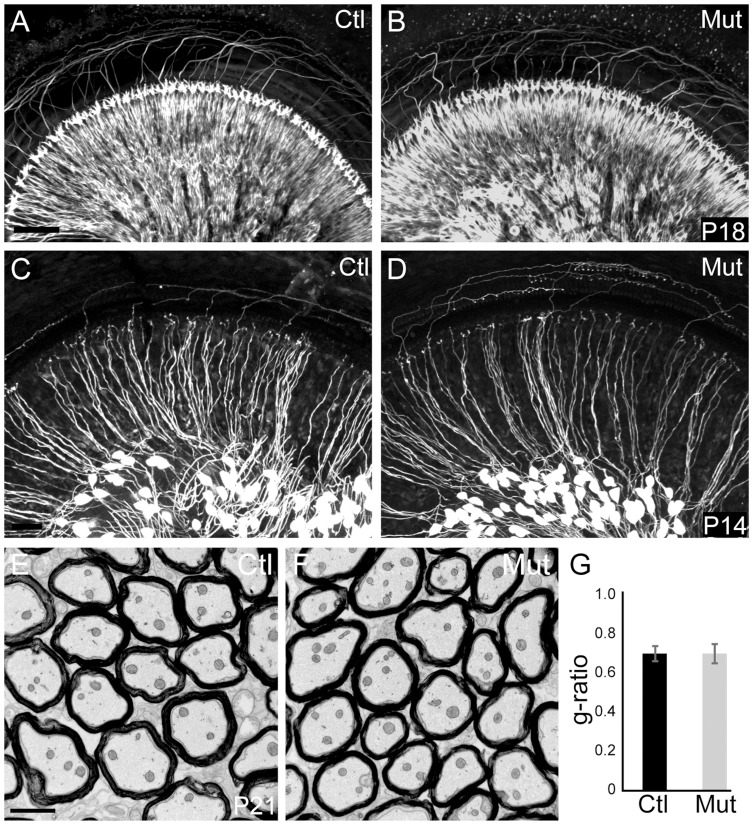
Peripheral SGN connectivity is normal in *Npr2* mutant mice. (**A,B**) Cochlear innervation was visualized by neurofilament immunostaining of P18 whole cochleae. Visual inspection revealed no obvious difference in the peripheral pattern of connections between wild-type control (Ctl, n = 2) (A) and *Npr2* mutant (Mut, n = 2) (B) animals. (**C,D**) SGN projections in the P14 cochlea were labeled by crossing *Neurog1-CreER^T2^* to the *AI14:tdTomato* reporter strain, which enables visualization of a subset of SGNs along the length of the cochlea. Individual SGNs of control animals (Ctl, n = 3 heterozygotes) (C) show neatly organized projections within the cochlea. Individual SGN processes of *Npr2* mutant (Mut, n = 5) cochleae (D) showed no qualitative differences compared to controls. Note that the degree of labeling can vary slightly independent of genotype, due to fluctuations in Cre activity. (**E**) Electron micrograph of a transverse section of myelinated SGN axons in the eighth nerve in a control P21 animal. (**F**) Similar electron micrograph of the eighth nerve of an *Npr2* mutant at P21 shows normal axonal diameters and normal myelination. (**G**) The g-ratio of *Npr2* mutants did not differ from controls (*P* = 0.87, Student's t-test) and was near optimal. Scale bars in A, 50 µm; C, 2 µm.

Previous studies established that Npr2 is absolutely required for bifurcation of sensory neuron axons with no obvious effects on the peripheral processes [24], [25], highlighting the utility of the *Npr2* mutant mouse as a model for central auditory wiring defects. However, the peripheral auditory system has not yet been examined. To investigate whether there are any obvious changes in the innervation of the mature cochlea, we evaluated the gross organization of SGN peripheral processes after the onset of hearing, which is at about postnatal day 12 (P12) in mice [31]. Neurofilament immunostaining revealed no obvious differences in the overall pattern of cochlear innervation at P18, with orderly arrays of radial bundles present in both *Npr2* mutants (n = 2) and controls (n = 2) (Fig. 1A,B). Moreover, individual SGN fibers, as visualized at P14 by crossing *Neurog1-CreER^T2^* to *AI14-tdTomato* reporter mice, exhibited normal morphologies, with single, unbranched peripheral processes extending directly towards the organ of Corti both in controls (n = 3) and mutants (n = 5) (Fig. 1C,D). Similarly, the SGN central processes in the auditory nerve peripheral to the bifurcation in the nerve root seemed normal in *Npr2* mutant mice at P21, as assessed by electron microscopy (Fig. 1E, F). The g-ratio, the ratio of the axon diameter to the total myelinated fiber diameter, did not differ significantly between control (n = 3) and *Npr2* mutant (n = 3) animals (*P* = 0.87) (Fig. 1G). In both groups, the observed ratio was near the optimal for conduction [32]. Hence, development of the peripheral auditory system appears normal in the *Npr2* mutant strain, which therefore offers a useful model for examining the anatomical and functional consequences of central auditory wiring defects.

### SGN projections are disorganized in *Npr2* mutant cochlear nuclei, with persistent defects in axonal bifurcations

**Figure 2:**
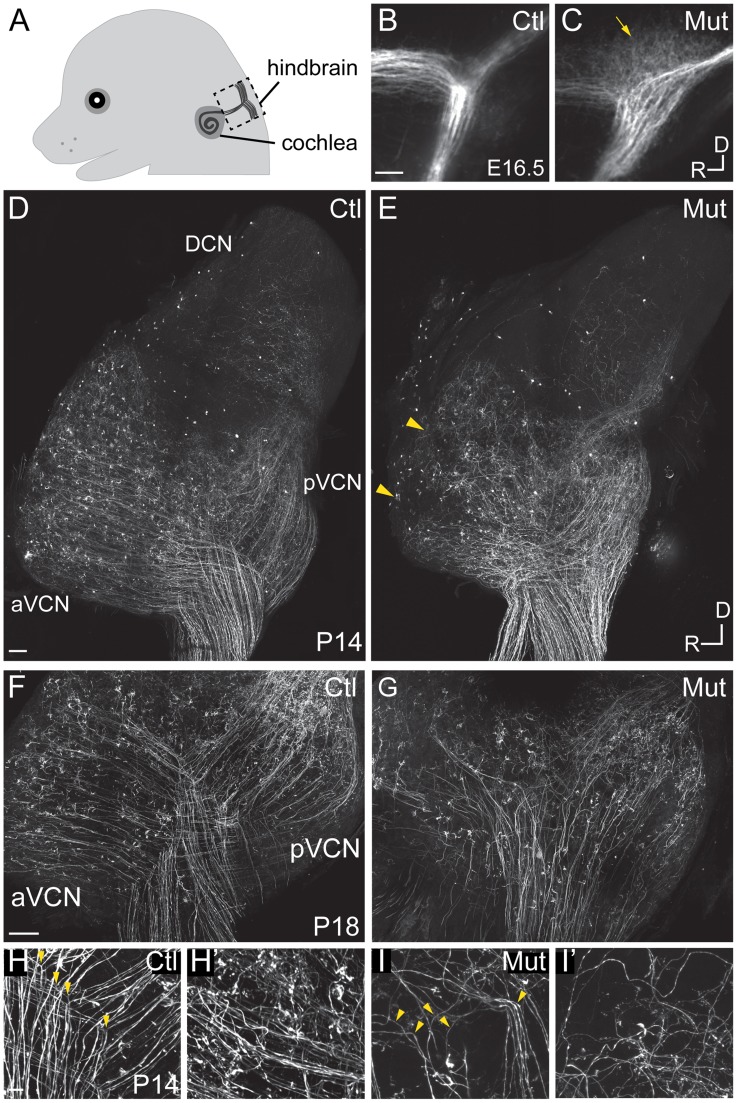
*Npr2* mutant mice show SGN central axon guidance and bifurcation defects. (**A**) Schematic diagram of E16.5 embryo head showing SGN axons projecting from the cochlea into the hindbrain. The boxed area indicates the hindbrain region shown in B and C. (**B,C**) Dye labeling of SGN central axons in the hindbrain at E16.5. (**B**) SGN axons normally exhibited a stereotyped bifurcation pattern in the developing brainstem at E16.5, as shown in a control heterozygous embryo (Ctl). (**C**) In *Npr2* mutants, axons appeared disorganized in the region where they would normally bifurcate (arrow), and the nerve root lacked the distinctive Y-shape. Dorsal (D) is up and rostral (R) is to the left. (**D–I**) Genetic labeling of SGN central axons at postnatal stages (P14–18) using *Ngn1-cre^ERT2^* and *AI14-tdTomato*, which allows random, relatively sparse labeling of SGNs. (**D–E**) Tiled confocal stack projections showing the entire cochlear nucleus of control (Ctl) (D) and *Npr2* mutant (Mut) (E) animals at P14, an approach that permits the overall pattern of SGN axon organization to be qualitatively assessed. Control SGN axons projected in a highly organized fashion to the aVCN, pVCN, and DCN (D). In an *Npr2* mutant, SGN axons still projected to aVCN, pVCN, and DCN, but in a disorganized pattern (E). Yellow arrowheads indicate regions in the aVCN that appear under-innervated. (**F–G**) Confocal stack projections of vibratome-sectioned control and *Npr2* mutant cochlear nuclei at P18. Visual inspection of SGN axons confirmed the presence of stereotypical Y-shaped branch points and orderly projections to aVCN and pVCN in controls (F). In contrast, SGN projections to aVCN and pVCN were disarrayed in *Npr2* mutants (G). (**H–H′**) Control SGN axons exhibited characteristic bifurcations (yellow arrowheads, H) and formed organized bundles of axonal branches in aVCN (H′) at P14. (**I–I′**) *Npr2* mutant SGN axons generally turned instead of bifurcating (yellow arrowheads, I) and followed aberrant trajectories in aVCN (I′). Scale bar in **A**, 50 µm. Scale bars in **D** and **F**, 100 µm. Scale bar in **H**, 10 µm.

Previously, we showed that *Npr2* is expressed in SGNs and is required for the bifurcation of central SGN axons at E12.5 [21], and independent studies have shown a complete absence of axon bifurcation in these and other sensory neuron populations in *Npr2* mutants [24], [25]. Although DRG bifurcation defects have been shown to persist and ultimately disrupt the functional connectivity of the mature spinal cord [24], the long term effects of the *Npr2* mutation for central auditory wiring remain poorly characterized. To determine whether SGN central axons acquire additional defects, we characterized *Npr2* mutants at E16.5, when both branches have formed and projected tonotopically within the developing cochlear nuclei (Fig. 2A) [33]. Lipophilic dye labeling revealed that in *Npr2* mutants (n = 4), the cochlear nerve root lacked the Y-shaped morphology typical of control animals (n = 4) (Fig. 2B,C), consistent with a persistent bifurcation defect. In addition, whereas SGN axons were neatly bundled both proximal and distal to the bifurcation of the nerve in control animals, *Npr2* mutant axons were disorganized in the region where they would normally branch (Fig. 2B, arrow), resembling the first exploratory SGN axons that reach the hindbrain at E11.5 in wild-type embryos [21].

We next examined SGN central projections in *Npr2* mutant animals between P14–P18, after the auditory circuit is fully formed and functional hearing has begun. To label SGN axons, *Neurog1-creER^T2^* mice were crossed to *AI14 tdTomato* reporter mice, resulting in offspring with tdTomato expression in a random subset of SGNs due to leaky Cre activity in the absence of tamoxifen. In *Neurog1-CreER^T2^;AI14* mice, tdTomato expression in SGNs is sparse enough that neuronal morphology can be examined, but is distributed uniformly along the length of the cochlea so that the overall pattern of SGN innervation throughout the intact cochlear nuclear complex can be visualized and qualitatively assessed in cleared tissue. In control animals (n = 5), auditory nerve fibers projected in a highly stereotyped and ordered manner to the aVCN, pVCN, and DCN (Fig. 2D). In contrast, SGN afferent innervation was consistently disrupted in all *Npr2* mutants (n = 6) (Fig. 2E). SGN axons were able to reach all three divisions, but exhibited several signs of disorganization that were not observed in controls. First, whereas control axons formed distinct bifurcations that aligned with each other within the nerve root, we could not recognize an obvious zone of bifurcation in *Npr2* mutants. Moreover, a closer look at sections through the cochlear nuclei revealed a severe disorganization of projections in the aVCN and pVCN in *Npr2* mutants (Fig. 2G) compared to controls (Fig. 2F). Control axons exhibited clear bifurcations in the VCN (Fig. 2H), resulting in neat bundles of axonal branches in the aVCN (Fig. 2H′). In contrast, *Npr2* mutant SGN axons generally turned instead of bifurcating (Fig. 2I) and often followed aberrant paths in the aVCN (Fig. 2I′). Additionally, some patches of the aVCN appeared to be underinnervated (Fig. 2E, arrowheads). Thus, loss of axon bifurcation and abnormal trajectories persist even after the onset of hearing in *Npr2* mutant mice.

**Figure 3:**
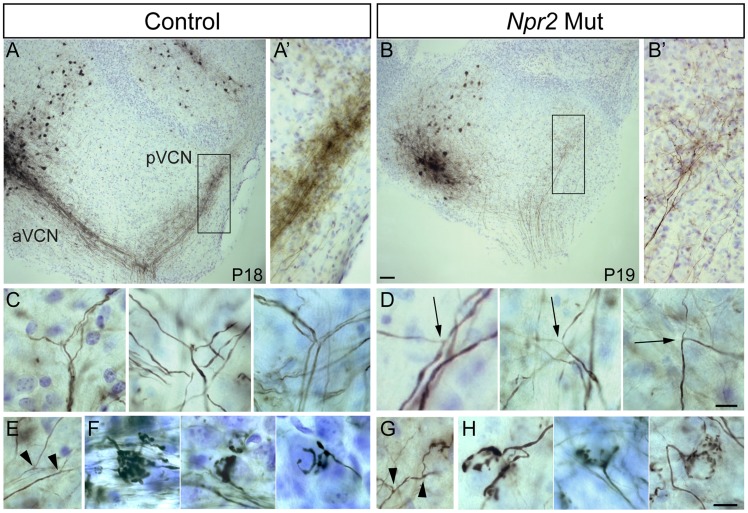
*Npr2* mutant SGN axons do not bifurcate properly but can form interstitial branches and morphologically normal synapses. (**A**) Injection of biocytin into the aVCN (left) in a parasagittal slice of the cochlear nuclei labeled fibers not only in the nerve root but also in the pVCN (right) in control (Ctl) animals at P18. (**A′**) A close up view of boxed region in **A** showing labeled descending branches of SGNs. (**B**) In an *Npr2* mutant (Mut) at P19, biocytin injection into the aVCN also labeled fibers in the nerve root and in the pVCN, showing that at least some fibers branched at the nerve root. (**B′**) A close up view of the boxed region in B shows labeled SGN fibers and terminals in the pVCN. (**C,D**) Magnified views of bifurcation zone in control (C) and mutant (D) animals. (C) Examples of stereotyped bifurcations in control animals. (D) In mutants, rare fibers that do branch in the appropriate region do so at irregular angles and with one thinner and one thicker branch (arrows). (**E–H**) SGN axons can still form interstitial branches and elaborate morphologically normal synaptic endings in *Npr2* mutants. Interstitial SGN axon branches, which were distinguished from bifurcations according to morphological criteria, are present in both control (E, arrowheads) and mutant (G, arrowheads) animals. Similarly, endbulbs of Held, which are one terminal whereby SGNs contact bushy cells, show the same types of branching patterns in the control (F) and *Npr2* mutants (H). Scale bars in **B**, 50 µm. Scale bars in **D** and **H**, 10 µm. Dorsal is up and rostral is to the left in all panels.

Although the overall pattern of innervation was clearly abnormal, SGNs projections were nevertheless present in all divisions of the *Npr2* mutant cochlear nuclei (Fig. 2E). Given that *Npr2* is required for axon bifurcation but not for development of collaterals [24], [25], we hypothesized that unbifurcated SGN axons might eventually form interstitial branches that are able to grow into other regions of the cochlear nuclei. To determine whether SGNs innervating the aVCN can still form branches that project to the pVCN, we labeled fibers with biocytin injections into the aVCN and searched for labeled fibers in the pVCN. In control animals (n = 49), such injections labeled the ascending branch retrogradely to the nerve root and they labeled the descending branch anterogradely through the pVCN and into the DCN (Fig. 3A). The labeled descending branches formed a tight bundle in the octopus cell area, where the SGN fibers converge on their way to the DCN (Fig. 3A′). In *Npr2* mutant animals (n = 28), obvious bundles were never seen. However, a few widely spread fibers in the octopus cell area were consistently labeled (Fig. 3B,B′), indicating that some individual SGN fibers managed to innervate both regions in spite of the axon bifurcation defect. Since there are no molecular markers to distinguish bifurcations from interstitial branches, we instead relied on morphological criteria to recognize bifurcations. Bifurcations are usually the first branch points within the cochlear nuclei, are found in a predictable location, and exhibit a characteristic Y-shaped morphology. No branch point in any of the 34 mutant cochlear nuclei in which biocytin was injected into the aVCN or into the cut end of the nerve displayed the morphology of normal bifurcations. Whereas in controls, the parent axon gave rise to two equally thick branches at roughly 120° angles (Fig. 3C), branches in the vicinity of the nerve in *Npr2* mutants exhibited more varied angles and one branch was often abnormally thin (Fig. 3D, arrows). Additionally, *Npr2* mutant SGN axons extended branches (Fig. 3G, arrowheads) that resembled the interstitial branches found in controls (Fig. 3E, arrowheads). Thus, it seems likely that the branches that SGN axons form in *Npr2* mutant mice are interstitial branches rather than true bifurcations. Nonetheless, axonal branches in the aVCN terminated in endbulbs of Held with the usual range of sizes and shapes in both controls (Fig. 3F) and mutants (Fig. 3H), indicating that despite their defective branching patterns and trajectories, *Npr2* mutant SGN axons are still able to find appropriate targets and make specialized synapses with normal morphology. Thus local interactions seem to be able to govern synaptogenesis independent of the changes in axon trajectory.

### Disruption of tonotopic organization in *Npr2* mutants

**Figure 4:**
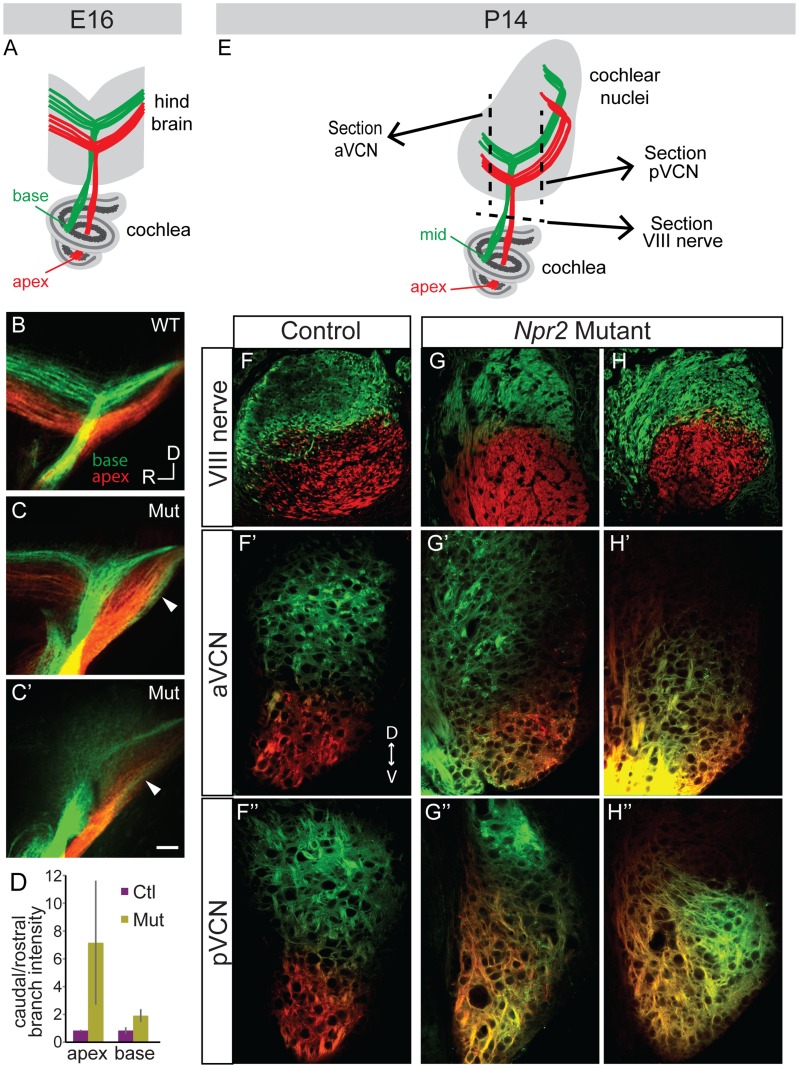
Tonotopy of SGN axons in the cochlear nuclei is blurred in *Npr2* mutants. (**A**) Schematic diagram illustrating tonotopic dye labeling at E16. Labeling of SGNs in the cochlea with red (apex) and green (base) lipophilic dyes allowed their relative positions to be traced to the hindbrain. (**B**) In a wild-type (WT) E16 embryo, fibers from the base and apex bifurcated in the nerve root in separate bundles. (**C–C′**) Similar labeling in two different *Npr2* mutants (Mut) shows that fibers from the base and apex were incompletely segregated in the hindbrain. The gross tonotopy was preserved, but some overlap in dye labeling was seen between axons from basal and apical SGNs (arrowheads). Scale bar, 50 µm. (**D**) The intensity of labeling of SGN axons revealed a caudal bias in *Npr2* mutants, with more axons projecting towards the developing pVCN than the aVCN (*P*<0.05, Student's t-test). (**E**) Schematic diagram illustrating tonotopic dye labeling at P14. The planes of sections illustrated in F–H are indicated by dotted lines. (**F–F″**) In a control animal, axons arising from the middle and from the apex of the cochlea were segregated in the auditory nerve and in the aVCN and pVCN, as assessed qualitatively using confocal imaging. (**G–G″, H–H″**) Two examples of *Npr2* mutants. Axons arising from the middle and apical turns of the cochlea were properly segregated in the nerve, indicating that the dyes labeled physically distinct populations of neurons as in controls (G,H). However, axons from these neurons were intermingled in the aVCN (G′, H′) and pVCN (G″, H″) of the same animals.

 Since the peripheral organization of SGN projections in the cochlea appeared unaffected in *Npr2* mutants, SGNs are predicted to receive sharply tuned frequency information from hair cells. However, given the disorganization of SGN central axons in *Npr2* mutants, we wondered whether mutant SGN axons preserve the tonotopic order of their projections as they exit the cochlea and find their way into the cochlear nuclei. Crystals of the lipophilic dyes, DiI and DiD, were inserted into the apical and basal turns of the cochlea in fixed E16.5 mouse heads and the dye was allowed to diffuse anterogradely through SGN axons to the hindbrain (Fig. 4A). In control animals (n = 2 wild-type and 2 heterozygote), SGN axons were tonotopically segregated within the eighth nerve, and their bifurcation points fanned out in tonotopic order within the developing cochlear nuclei, with axons from more basal SGNs bifurcating more dorsally than apical SGNs (Fig. 4B). The gross tonotopic segregation observed in control embryos was maintained in *Npr2* mutants (n = 4) (Fig. 4C,C′). However, in some *Npr2* mutant embryos (n = 2/4), intermingling of apical and basal projections was observed (Fig. 4C,C′, arrowheads), suggesting imprecise tonotopy. Additionally, mutant axons appeared to project more strongly towards what will become the pVCN and DCN, quantified by comparing the fluorescence intensity of the branches projecting rostrally vs. caudally (*P*<0.05) (Fig. 4D). Apical SGNs were more strongly affected than basal SGNs and showed a stronger bias towards the developing pVCN and DCN.

To determine whether the blurring of tonotopy persists through the onset of hearing, similar dye labeling of SGNs was performed at P14 by placing DiI and DiD crystals in the apical and mid-turns of the cochlea, respectively (Fig. 4E). In controls (n = 2 wild-type), clear segregation of the two dyes was observed in the eighth nerve (Fig. 4F) and this segregation was maintained both in the aVCN (Fig. 4F′) and pVCN (Fig. 4F″), as assessed using confocal imaging. In *Npr2* mutants (n = 4), the axons from apical and mid-turn SGNs were also appropriately segregated within the eighth nerve (Fig. 4G, H), confirming that the dyes labeled distinct populations of neurons in the cochlea. However, the projections overlapped extensively in the aVCN (Fig. 4G′, H′) and/or pVCN (Fig. 4G″, H″). Some overlap was apparent in all of the mutants; variability in precise size and location of the dye crystals prevented quantification of the degree of mixing. Thus, tonotopic segregation appears normal in the auditory nerve, but is degraded within the cochlear nuclei of *Npr2* mutants.

**Figure 5:**
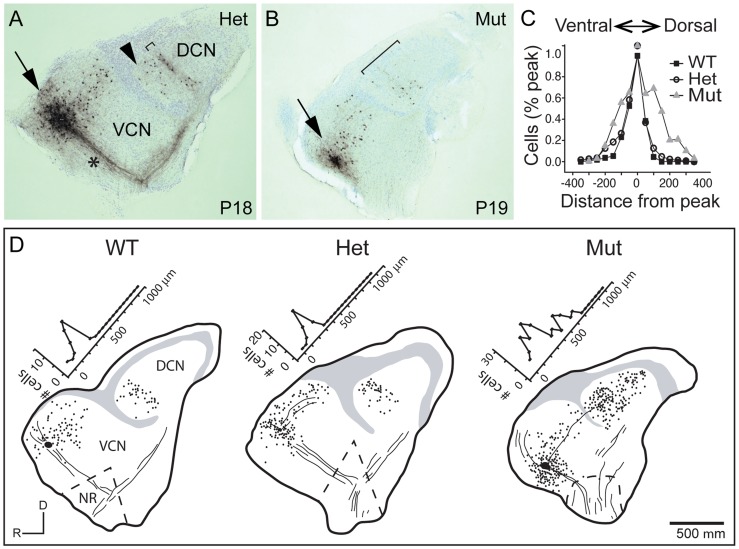
Tonotopy of TV neuron projections from the DCN to the aVCN is also blurred. (**A**) Photomicrograph of a section of a parasagittal slice from a P18 heterozygote control animal with a biocytin injection into the aVCN (arrow). Biocytin labeled processes and cell bodies of neurons that passed through the injection site. Labeled processes included auditory nerve fibers (asterisk), the axons and terminals of TV cells whose cell bodies lie in the DCN (bracket), and the dendrites and local axonal collaterals of T stellate and D stellate cells whose cell bodies formed a halo around the injection site. Labeled TV cells clustered in a band (bracket) among terminals of auditory nerve fibers that were labeled by the same injection, showing that the TV cells lie in the same isofrequency lamina as their VCN targets. A few TV cells whose axons crossed the injection site on their way to more ventral regions were labeled ventral to (arrowhead) the labeled band of TV cells. No cell bodies were labeled in the DCN dorsal to the labeled band, indicative of the sharp tonotopic organization of the projection. (**B**) A similar injection of biocytin into the aVCN of a P19 *Npr2* mutant animal labeled a more diffuse bundle of auditory nerve fibers, a halo of neurons in the aVCN, and TV cells that were more scattered than in the heterozygote (bracket). (**C**) To compare the distribution of labeled neurons between control and mutant animals, peaks of distributions were lined up and normalized. In WT (n = 6 slices, 258 cells) and Hets (n = 20 slices, 914 cells), labeled cells were distributed in a sharp band, with no labeled cells more than 150 µm from the peak on the dorsal side. Since no clear bands were observed in *Npr2* mutants (Mut) (n = 11 slices, 858 cells), histograms were aligned along their medians, at which half of the labeled cells lay more dorsal and half more ventral. The bands were sharp in both WT and Het animals, but were significantly broader in *Npr2* mutant mice (*P*<0.001, ANOVA). No differences were detected over the age range examined between P14 and P26. (**D**) Examples of reconstructed slices, with labeled cells marked with dots, gray regions denoting areas containing granule cells in the largest section, and lines indicating the location of some of the labeled fibers. Numbers of labeled TV cell bodies were plotted as a function of distance along the tonotopic axis of the DCN, as illustrated. Scale bar, 500 µm.

The abnormal tonotopic organization of SGN projections raised the question of whether intrinsic neuronal circuits within the cochlear nuclei are similarly disrupted. Tuberculoventral (TV) cells are glycinergic neurons that reside in the deep layer of the DCN and innervate targets in the aVCN and pVCN, forming a negative feedback circuit. They are tonotopically arranged, receiving input from the same auditory nerve fibers as their targets and therefore exhibit similar tuning [1], [2], [34]. The pattern of TV cell connectivity was examined by injecting biocytin into the aVCN, which normally labels TV cell bodies in the DCN as well as SGN afferent fibers that project to that isofrequency band [1] (Fig. 5A–B). Labeling follows the tonotopic organization of the cochlear nuclei: dorsal injections labeled bands of TV cells dorsally in DCN, whereas ventral injections labeled TV cells in a more ventral position in the DCN. In control animals (n = 16 wild-type and 33 heterozygote), a few labeled cells in the DCN were located ventral to the isofrequency band, because their axons crossed the injection site to innervate more ventral regions of the aVCN (Fig. 5A, arrowhead). However, labeled cells dorsal to the band in the DCN were not observed in normal animals, indicative of the sharp tonotopic organization (Fig. 5A,D). In contrast, in *Npr2* mutant mice (n = 28), the labeled cell bodies were found over a large span of the DCN, even when the injections were made ventrally in the aVCN (Fig. 5B, D). To quantify this result, the distribution of labeled cells along the tonotopic axis was measured in reconstructions of 37 slices with injections into the ventral half of aVCN (Fig. 5C). In control mice (n = 26 cochlear nuclei from 12 heterozygote and 4 wild-type mice), the distribution of labeled cells aligned at their peaks showed a sharp peak that tapered ventrally toward the granule cell lamina, with an average half-width of 132±80 µm. In *Npr2* mutants (n = 11 cochlear nuclei from 7 mice), the distributions lacked sharp peaks. The average distribution of labeled cells, aligned on the median, was significantly broader, with an average half-width of 276±130 µm (*P*<0.001), reflecting the more diffuse organization observed within individual cochlear nuclei. These findings indicate that the tonotopic organization of the TV cell projection in mutant cochlear nuclei is less precise than in control animals, consistent with the overall disruption in SGN axon topography shown by genetic and dye labeling.

### The abnormal wiring pattern in *Npr2* mutants does not noticeably affect auditory brainstem responses (ABRs)

**Figure 6:**
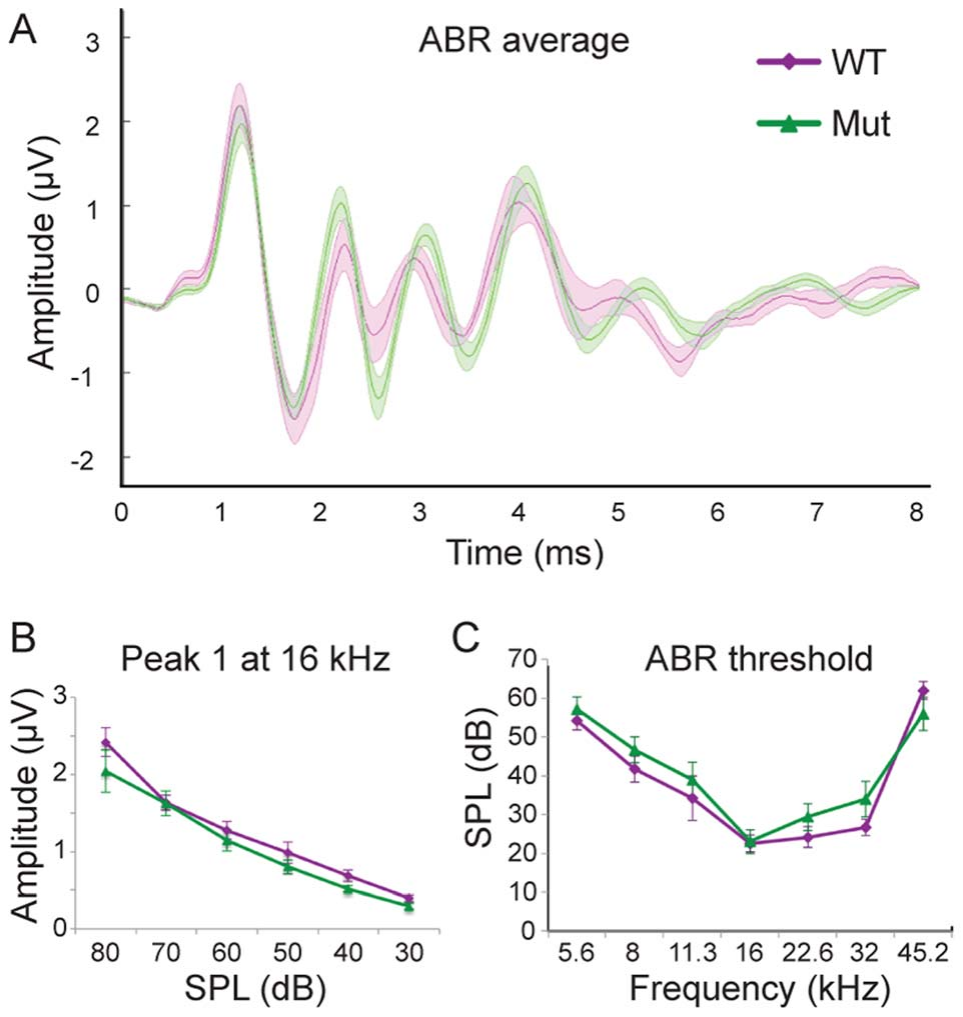
*Npr2* mutants show normal auditory responses. (**A**) Average ABR waveforms at 16 kHz for wild-type (WT) (purple, n = 6) and *Npr2* mutant (Mut) (green, n = 15) animals. The average is shown by the dark lines, and the shaded areas show the standard error of the mean. (**B**) Wave 1 reflects the synchronous firing of auditory nerve fibers. Its amplitude decreased with sound level similarly in WT (purple) and Mut (green) (*P*>0.3 at all frequencies, Student's t-test). (**C**) Average ABR thresholds for WT (purple) and *Npr2* mutant (green) animals across frequencies. No significant difference was observed between WT and *Npr2* mutants (*P*>0.3, Student's t-test).

Our anatomical studies show that although the innervation of the cochlea is not altered noticeably, there is a consistent and striking change in SGN central axonal innervation patterns in *Npr2* mutant mice. While changes in the periphery are well-known to diminish auditory sensitivity, how a loss of precision in the organization of SGN inputs to the cochlear nuclei might affect hearing is unclear. To address this question, we compared auditory brainstem responses (ABR) in six-week old wild-type and *Npr2* mutant mice. ABRs are generated by the synchronous firing of groups of aligned axons. In cats, the first large positive and negative waves reflect the firing of axons of SGNs, the second positive wave reflects the firing of neurons in the VCN that lie near the nerve root, the third positive wave reflects activation of the VCN rostral and caudal to the nerve root and the superior olivary complex, and later waves reflect the summation of activity at many stages of the auditory pathway [35]. Similar waveforms are observed in mice; it is broadly accepted that the first two peaks reflect activity in the nerve and cochlear nuclei as in cats [36], [37]. No significant difference was observed between control (n = 6 wild-type) and *Npr2* mutant (n = 15) mice in the shape or amplitude of the early peaks in responses to 16 kHz tones, which activate the most sensitive regions of the cochlea in mice [38] (*P*>0.3 for the amplitudes of peaks one and two at all sound pressure levels) (Fig. 6A, B). The normal average ABR waveforms confirm the absence of obvious peripheral defects and suggest that the timing of firing of SGNs and of their targets in the VCN is also apparently normal. In addition, ABR thresholds did not differ significantly between wild-type and *Npr2* mutant mice (*P* = 0.38) (Fig. 6C). Thus, within the resolution of these measurements, the sensitivity and timing of firing of auditory neurons in the brainstem seem normal in *Npr2* mutants.

It should be noted that *Npr2* mutant mice exhibit additional abnormalities, including dwarfism and cardiac deficits [39] that compromise their health and often cause them to die within the first postnatal month. Thus, it is possible that no obvious ABR phenotype was observed because the animals that survived to the testing stage were the healthiest and least abnormal. However, cochlear nuclear innervation defects were fully penetrant and varied only in severity. Moreover, since it is difficult to establish behavioral baselines in these animals, we were unable to use pre-pulse inhibition of the acoustic startle reflex to test for deficits in specific hearing tasks, such as frequency discrimination, gap detection, and sound localization.

### Auditory signal transmission has many normal features in *Npr2* mutants

Although ABRs did not reveal any significant differences in auditory responsiveness in *Npr2* mutant mice, this method assesses the overall activity of the population, leaving open the possibility that individual cells may not transmit signals normally. To determine whether *Npr2* mutant axons are indeed able to develop normal synapses despite the change in branching patterns and trajectory, we made intracellular recordings in slices. Cochlear nuclear neuronal responses to sound depend on the pattern of convergence of synaptic inputs, the physiological properties of those inputs, and the electrical properties of target neurons that shape the voltage responses to synaptic currents. Whole-cell patch recordings in slice preparations of the cochlear nuclei confirmed that the three principal cell types of VCN (bushy, octopus, and T stellate cells), recognizable by the differences in their intrinsic electrical properties, are present in *Npr2* mutants. In *Npr2* mutants as in control animals, bushy and octopus cells fire transiently in response to depolarizing current pulses, whereas T stellate cells respond with trains of action potentials that last for the duration of the depolarization, in both wild-type and mutant animals [10], [12], [40], [41] (S1 Figure). Comparison of wild-type (n = 3) and mutant (n = 4) bushy cell properties revealed no change either in the resting potential (−65±1.7 mV in wild-type vs. −66±2.1 mV in *Npr2* mutant) or input resistance (92±8 MΩ in wild-type vs. 97±9 MΩ in *Npr2* mutant); the properties of T stellate (n = 2) and octopus cells (n = 2) were also within the normal range. The absence of any measurable differences in the intrinsic properties indicates that mutant neurons are capable of signaling as rapidly and precisely as the wild type.

**Figure 7:**
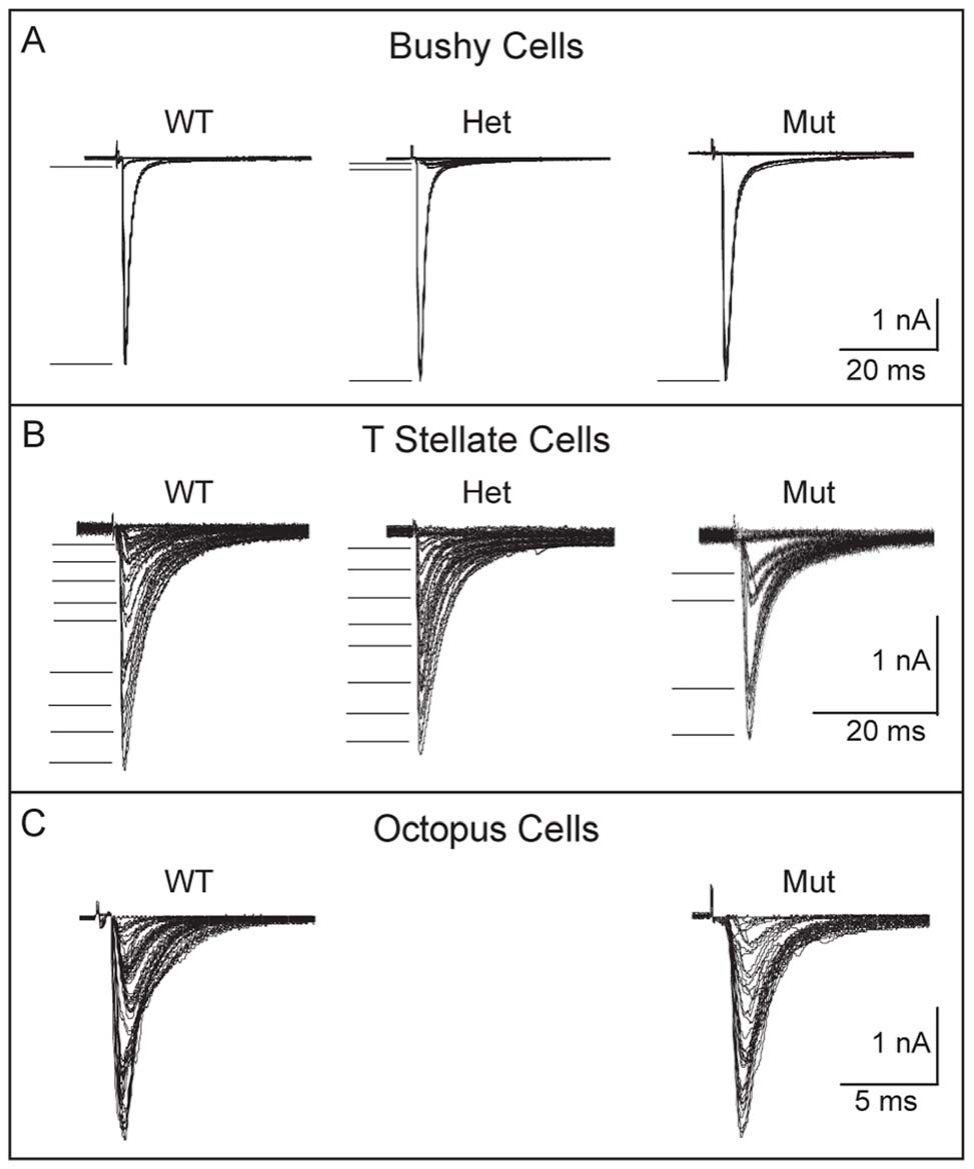
Convergence of SGNs onto bushy and T stellate cells tends to be lower in *Npr2* mutants. (**A**) Voltage-clamp recordings (−65 mV) from individual bushy cells showed that in wild-type and heterozygote controls, gradual increase in the strength of shocks applied to auditory nerve fiber bundles evoked first one or two small jumps in current, presumably from bringing to threshold one or two fibers that contacted the recorded bushy cells, and then a large jump likely from bringing to threshold a fiber that contacted the bushy cell with an endbulb of Held. In the mutant, the response was all-or-none, increasing in a single step. In all genotypes, at least one of the steps was >1 nA. There was no obvious difference in the amplitudes of steps between mutant, heterozygote and wild type mice (P17–19). Five out of seven mutant bushy cells likely received one input through an endbulb. (**B**) Recordings from individual control T stellate cells showed that the synaptic current grew in 8 or 9 small steps, but that in a T stellate cell recorded anteriorly in an *Npr2* mutant, the response grew in only 4, larger steps. Small numbers of steps were recorded in 4 of 10 mutant T stellate cells, all of which lay anteriorly; the remaining 6 cells were located posteriorly. This difference is statistically significant (*P*<0.001, Student's t-test). (**C**) Recordings from individual octopus cells grew in more steps that were too numerous to count both in the wild type and in mutants. There was no discernible difference between them.

Another important determinant of acoustic signal transmission is the number of SGN inputs that contact each target neuron, which ranges from few (for bushy and T stellate cells) to many (for octopus cells). Given the abnormal trajectories of SGN axons seen within the cochlear nuclei, we asked whether SGNs would still converge normally on principal cells of the VCN in *Npr2* mutants. The number of excitatory inputs that converge on a recorded cell can be estimated by measuring the growth of synaptic responses to shocks of fiber bundles as the shock strength is gradually increased because the synaptic response grows in steps as additional fibers are brought to threshold [16]. The number of steps in the increase in synaptic current is thus an estimate of the number of excitatory inputs. Bushy cells receive converging input from a small number of SGNs. In the mutants, as in control animals, some jumps were small and others were large, reflecting the fact that bushy cells in mice receive input from small bouton endings as well as large endbulbs of Held [7], [16], [42] (Fig. 7A). The number of converging inputs to bushy cells in *Npr2* mutants fell into the normal range, between 1 and 6 [16]. However, a surprisingly large proportion had only a single input (5/7 in *Npr2* mutants, compared with 5/21 similar bushy cells in a wild type strain [16]. Interpretation of these findings is complicated by the fact that there are multiple types of bushy cells: globular bushy cells that project to the medial nucleus of the trapezoid body and spherical bushy cells that project to the lateral or medial superior olivary nuclei. The sole electrophysiological distinction between these cell types in slices is the number of converging inputs [7], [16]. Especially when the aVCN is disorganized, it is impossible to know whether populations of different types of bushy cells were sampled equally. However, our results suggest that bushy cells in the aVCN in *Npr2* mutants likely receive input from fewer SGNs than normal.

To see whether other target neurons in aVCN also receive fewer inputs, we performed a similar analysis of T stellate cells, which are present both in aVCN and pVCN. Shock-evoked synaptic responses in T stellate cells normally grow with between five and eight steps, each delivering roughly equal steps of current of between 100 and 300 pA [16], [43] (Fig. 7B). Many of these responses are likely to arise from SGNs but some could also arise from other T stellate cells [43]. In control mice, no differences have been reported between T stellate cells in pVCN, where they are most abundant, and in the aVCN [40], [44], [45]. In *Npr2* mutants, 6/10 of the T stellate cells we recorded were in the pVCN, near the octopus cell area. Convergence of inputs in these cells was normal, averaging 7.5±1 (n = 6). In contrast, in the 4/10 T stellate cells that were recorded more anteriorly, evoked responses grew in significantly fewer current steps (3.5±0.6, n = 4) (*P*<0.001) (Fig. 7B).

Octopus cells reside in pVCN and would therefore not be expected to show a similar change in the number of SGN inputs. However, these cells are so heavily innervated by SGNs that it is not possible to estimate the actual number from the growth of synaptic responses with shock strength [12], [13]. Instead, the synaptic responses generally grow in steps so small that the growth appears graded. In *Npr2* mutant mice, the growth of synaptic responses showed more irregularity than we have observed in CBA or ICR mice [12], [16], but this irregularity was also observed in control mice. No differences in convergence between wild-type and mutant mice could be resolved in octopus cells (Fig. 7C).

Together, these data suggest that in *Npr2* mutants, convergence of SGNs onto bushy and T stellate cell targets in the aVCN is reduced, while in the pVCN, convergence onto T stellate cells and octopus cells is normal. This subtle change in circuit organization is consistent with our finding that SGN projections are biased towards the pVCN and DCN at embryonic stages.

**Figure 8:**
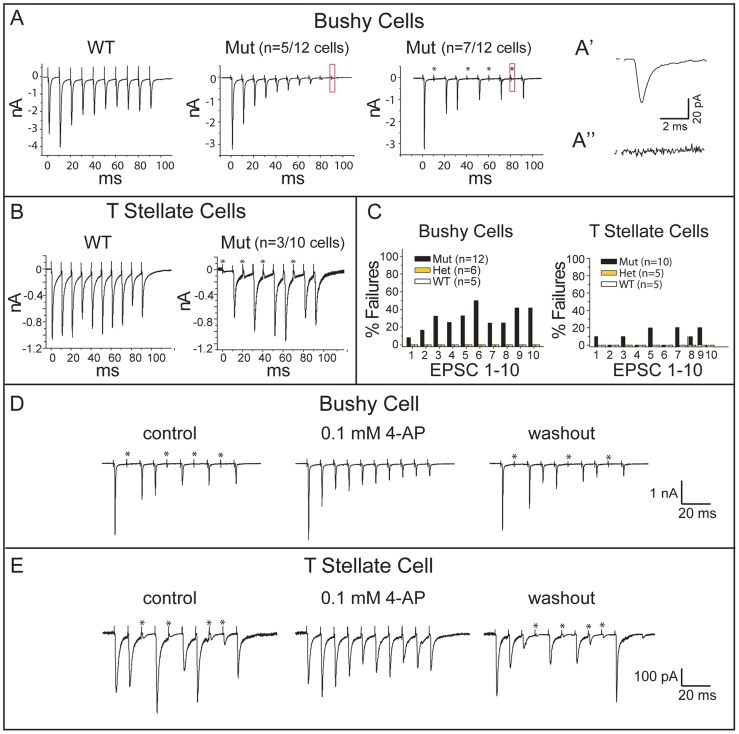
*Npr2* mutants exhibit normal synaptic depression but abnormal intermittent synaptic failures. (**A**) Trains of shocks delivered at 100 Hz evoked synaptic responses that tended to diminish in amplitude, confirming synaptic depression in both control and *Npr2* mutant animals. However, some mutant bushy cells failed to respond to some of the shocks in the train. (**A′**) The small response (boxed) in the middle panel is shown at higher magnification to show that this cell had strong synaptic depression but that every shock evoked a synaptic current. 7 of 12 bushy cells showed synaptic failures such as those shown in the right panel. (**A″**) At higher magnification, no response was detected in the boxed region, indicating that this was a synaptic failure. (**B**) In T stellate cells, trains evoked responses with less synaptic depression than in bushy cells in the WT as well as in mutants. Failures in transmission were observed in mutant but not in WT animals. (**C**) Quantification of EPSC failures in control (WT and Het) and *Npr2* mutant (Mut) bushy and T stellate cells in response to the first 10 shocks in a train. No failures were observed in WT or Het cells, whereas in *Npr2* mutants, failures sometimes occurred, even in responses to the first shock. (**D, E**) Synaptic failures in *Npr2* mutants were reversibly eliminated by 4-aminopyridine (4-AP). In a bushy cell (**D**) and in a T stellate cell (**E**), failure to evoke EPSCs was reversibly abolished by 0.1 mM 4-AP. Blocking K^+^ channels heightens and lengthens action potentials, making them less susceptible to conduction block.

To determine whether the observed changes in the pattern of connectivity are accompanied by changes in the nature of transmission between SGNs and their cochlear nuclear targets, we examined the pattern of synaptic responses to trains of shocks. In wild-type mice, repeated stimulation of the auditory nerve consistently evokes synaptic responses, although when driven at high rates, synaptic responses show depression, with a stronger effect in bushy than in T stellate cells [15], [16], [46]. Synapses between SGNs and principal neurons in the VCN in *Npr2* mutants (22 cells from 22 animals) exhibited the expected synaptic depression observed in wild-type and heterozygous animals (21 cells in 10 wild type and 11 heterozygote mice) (Fig. 8A, A′). However, *Npr2* mutants differed from control animals in that shocks intermittently failed to evoke any responses in some neurons. For instance, in 7/12 bushy cells, some of the shocks in a train failed to evoke a response (Fig. 8A). Failures were sporadic and complete, with no synaptic response at all in the target neuron (Fig. 8A″). A similar phenotype was also detected in T stellate cells (Fig. 8B), with 3/10 cells sporadically failing to respond to shocks; in contrast, 0 of 10 wild type and heterozygote responses failed. One reason failures may have been detected in relatively fewer T stellate cells than in bushy cells is that failures of small inputs are difficult to detect. Indeed, in T stellate cells failure was often incomplete in that small (<10%) synaptic current remained, presumably because the larger of two inputs failed while the smaller one did not.

For both bushy and T stellate cells, failures occurred even after the first shock in the train, when depletion of neurotransmitter is not an issue (Fig. 8C). Together with the all-or-none character of the failures, these findings suggest that action potentials sometimes fail to invade the SGN synaptic terminals. To test whether a conduction block could be overcome by making action potentials in the parent axon taller and/or wider, we applied a low concentration of 4-aminopyridine (4-AP), a non-specific blocker of K^+^ channels used to relieve conduction block in patients with multiple sclerosis [47]. Indeed, 0.1 mM 4-AP eliminated synaptic failures reversibly in both bushy and T stellate cells (Fig. 8D, E). These results support the idea that *Npr2* mutant auditory nerve axons suffer from blocks in action potential conduction. Importantly, the responses that did occur showed normal, precise temporal tracking of inputs (Fig. 7). Thus, our data indicate that *Npr2* mutant mice exhibit altered spatial organization of the auditory circuit and less reliable action potential conduction, yet still maintain the overall temporal precision of auditory signal transmission.

## Discussion

The sense of hearing depends on accurate transmission of frequency and timing information from the cochlea to the brain by SGNs. Hence, SGN projections are organized spatially according to frequency and form synapses that preserve the timing of sound stimuli. Using a combination of anatomical and physiological methods, we find that these two fundamental characteristics are differentially affected in *Npr2* mutant mice, which exhibit blurred tonotopy in the cochlear nuclei but still form functional connections with largely normal electrophysiological features. Although there is a slight reduction in the convergence of inputs and occasional failures in transmission, the timing of neuronal firing at a population level and sound detection thresholds, as assessed by ABR, do not differ significantly between *Npr2* mutant and control mice. Taken together, these data indicate that central auditory circuits with defective spatial organization are still capable of normal signal transmission and hence auditory responsiveness, though it is unlikely that auditory processing in *Npr2* mutant mice is entirely normal. These findings highlight the importance of *Npr2* for central auditory circuit assembly and underscore the challenges of understanding the genetic basis of central auditory processing disorders.

### Disruption of SGN wiring patterns in *Npr2* mutants during development

Although recent studies have uncovered a number of genes required for cochlear wiring [48], how SGN central axons navigate to the cochlear nuclei is poorly understood. SGN axons reach the hindbrain and start to bifurcate by E12 in mice [21]. When the aVCN is not present, SGN axons still project to the brainstem and bifurcate [49], likely because the Npr2 ligand CNP is expressed along the entire rostral-caudal axis of the hindbrain [23]. In addition, since SGN axons enter the hindbrain at the level of rhombomere 4, which gives rise to the pVCN and DCN [50], this region may provide attractive cues that are primarily responsible for early SGN guidance decisions. Indeed, we find that *Npr2* mutant axons preferentially extend towards the developing pVCN and DCN, indicating that when required to make a directional choice without bifurcating, SGN axons show a caudal bias. Nevertheless, their projections follow aberrant trajectories, suggesting that *Npr2* is also required for normal responsiveness to cues in the environment. A direct role for Npr2 in axon guidance has not been clearly shown; although *Npr2* mutant DRG axons make occasional guidance errors upon entering the spinal cord, they follow grossly normal paths towards targets in the dorsal and ventral horns [24]. Moreover, expression of CNP is restricted to the dorsal neural tube embryonically [23], [25], making it improbable that a CNP-Npr2 interaction directs growth of SGN axons deeper within the developing cochlear nuclei. It therefore seems more likely that the observed guidance defects are secondary to the loss of bifurcation.

After bifurcating, developing SGN axon branches must navigate towards distinct regions of the cochlear nuclei while retaining the tonotopic organization established in the cochlea. This fundamental feature of the auditory pathway is established during embryogenesis [33], [51] and does not require hearing, although it is later refined by activity-dependent mechanisms that need not be driven by sound [52], [53]. We still know very little about the molecular mechanisms that direct this critical feature of central auditory circuit assembly, and the few examples of mutant mouse strains with disrupted tonotopy are complicated to interpret. For example, tonotopy is abnormal in mice lacking the transcription factor Neurod1, which acts early during SGN development [54]. However, in these animals, auditory afferents are intermingled with projections from the vestibular endorgans, suggesting that disrupted tonotopic organization is secondary to a general change in neuronal identity. Eph/Ephrin signaling may play a more specific role in central topographic projections, with *ephrin-B2* mutants exhibiting abnormally broad frequency bands in the DCN [55]. However, since EphA4/ephrin-B2 signaling is also involved in bundling of peripheral SGN projections extending towards the organ of Corti [56], the change in frequency responses in these mutants could also arise from peripheral disorganization.

*Npr2* mutant mice are especially interesting because defects in the tonotopic organization in the cochlear nuclei occur without obvious defects in cochlear organization. SGN axons are topographically ordered in the eighth nerve in *Npr2* mutants, but exhibit disorganization in the nerve root and blurred tonotopy in the cochlear nuclei, indicating that trajectories become disarrayed as they enter the auditory brainstem. The phenotype was fully penetrant, with abnormal topography apparent in genetically labeled SGNs, by labeling of the SGN axons with lipophilic dyes, and by biocytin labeling of second order neurons in the cochlear nuclei. Since SGN bifurcation points are tonotopically ordered within the nerve root, with small bundles of SGN axons bifurcating together, it is possible that the abnormal guidance of *Npr2* mutant axons exiting the eighth nerve disrupts this bundling, thereby perturbing the local tonotopic order. Indeed, proper fasciculation during axon guidance is known to play a key role in topographic mapping of axons in other systems [57], [58]. Alternatively, mutant axons may be unable to detect guidance cues in the environment, perhaps because key receptors are not trafficked properly to the branches that do form. In fact, tonotopy worsens over time, with only a few misrouted axons at E16.5 but apparent overlap at P14, as would be expected if the primary problem is defective guidance of the collaterals that sprout from the unbifurcated axons. Higher resolution labeling methods will be needed to discern whether individual fibers extend their primary axons to the tonotopically appropriate location, with blurring due to abnormal guidance of collaterals outside of this area. It is tantalizing to consider whether the disorganization of TV cell projections might also be secondary to the changes in SGN axon branching patterns. However, although expression is initially restricted to sensory neurons [24], [25], *Npr2* also appears to be transcribed in the cochlear nuclei later in development [59] (Allen Mouse Brain Atlas) and could act independently in other populations of neurons. Analysis of cochlear-specific *Npr2* conditional knock-outs will be necessary to resolve this issue.

### Functional consequences of abnormal innervation of the cochlear nuclei

Unexpectedly, the loss of tonotopic organization in the central auditory circuits of *Npr2* mutant mice results only in subtle changes in auditory function. ABRs, which are generated by the summation of coherent currents and thus largely reflect synchronous firing in bundles of axons [35] are normal in the mutants; intracellular recordings, which assay transmission to individual post-synaptic targets, show that evoked excitatory postsynaptic responses are normal when present but occasionally fail. The absence of any obvious ABR defect in *Npr2* mutants is consistent with our anatomical studies, which revealed no abnormalities in peripheral wiring, myelination, axon diameter, or synaptic morphology. It is also consistent with whole-cell patch-clamp recordings from individual neurons, which show that the principal cells of the VCN in *Npr2* mutants retain the ability to signal rapidly and with temporal precision. Indeed, the basic features of synaptic transmission were unaffected; EPSC amplitudes, kinetics, depression, and delays in the VCN of *Npr2* mutants were in the normal range [16]. The only salient defect observed was an occasional failure in transmission, which would not be expected to alter the timing of signaling in the population of neurons. Moreover, since amplitudes of the first wave did not differ significantly between mutants and controls, either roughly similar numbers of neurons are activated or the smaller heads of mutants compensate for slightly reduced numbers of active neurons. Normal ABRs are also observed in animals in which reorganization of central tonotopic maps is induced by persistent, moderate noise [60]. While this might mean that ABRs are not sensitive enough to detect such changes, it may also reflect the plasticity of central auditory circuits, as has also been described by others [52]. Overall, these physiological studies suggest that *Npr2* mutant SGNs are still able to respond to sounds with normal sensitivity and timing, despite the disrupted spatial organization. Thus, in contrast to what has been observed for development of the calyx of Held [17], functionally normal synapses can form even when SGN axons follow abnormal paths.

Although our physiological tests revealed no significant change in auditory responsiveness, it is still unclear whether the blurring of spatial organization represents a functional blurring at the level of frequency discrimination in *Npr2* mutants. In wild-type animals, the tuning of bushy and T stellate cells shows similar sharpness to that of auditory nerve fibers [11], indicating that SGNs that converge onto a single target neuron are similarly tuned. Since frequency coding is likely intact in the cochlea, activity-dependent synapse elimination, not only in the aVCN but also in TV cells of the DCN, could select for appropriate inputs with similar tuning in *Npr2* mutant mice even when they are not in the correct spatial location. Thus, the broadening of TV cell isofrequency mapping in *Npr2* mutants might reflect appropriate functional connections between cohorts of neurons that transmit similar frequency information, but that are no longer spatially confined to a tight band due to the disorganization of SGN afferents. Although pre-pulse inhibition of the acoustic startle response can be used in mice to test for frequency discrimination [61], such experiments are not possible with *Npr2* mutants, which have dwarfism and cardiac defects, and therefore await generation of *Npr2* conditional knockout animals.

Although the basic features of synaptic transmission were unaffected by the loss of Npr2 function, auditory signal transmission became less reliable, with some shocks to SGNs failing to produce any response in post-synaptic targets. Since the failures sometimes occurred in the first response of a train, they could not have resulted from the depletion of neurotransmitter. Furthermore, EPSC failures were all-or-none, indicating that some action potentials did not reach the SGN terminals. Additionally, *Npr2* mutant axons showed no significant loss of myelin and did not exhibit signs of the increased spike latency or jitter associated with dysmyelinated SGNs [62]. Given the changes in SGN axon branching patterns in *Npr2* mutants and our ability to reverse failures with a K^+^ channel blocker that strengthens action potentials, it is likely that failure occurred at branch points, which have long been recognized as being weak points in conduction [63]. Although a similar functional phenotype has not yet been described in other sensory neurons in *Npr2* mutants, DRG neurons do exhibit mildly impaired ability to activate target neurons upon capsaicin treatment [24], indicating the need for a more detailed analysis of these neurons.

Overall, our results suggest that the development of the auditory circuit is robust enough that surprisingly normal synaptic connections can be made even in the face of disorganized topography. It is unlikely that *Npr2* mutant mice have completely normal hearing, but more subtle behavioral tests will be required to reveal deficits. Notably, it is estimated that ∼1% of people with normal hearing sensitivity, and therefore normal cochlear function, have defects in their ability to process sound [64]. Unambiguously identifying and characterizing patients with these central auditory processing disorders has been challenging, because multimodal sensory, language, and attention deficits can accompany or mimic central auditory processing disorders, thereby complicating diagnosis [65]. Interestingly, *NPR2* mutations cause achondroplasia in humans [30], suggesting that closer examination of auditory function may be warranted in such patients. The identification of central wiring defects in *Npr2* and other mutant mouse strains may lead to more directed clinical analysis of hearing in human patients carrying analogous mutations and therefore improve the diagnosis and classification of these disorders in the future.

## Materials and Methods

### Mice

The following mouse strains were used: *Neurog1-creER^T2^* mice [33], *AI14-tdTomato* mice (Jackson Laboratories, Stock Number 007908), and *Npr2^cn^* mice which carry a missense point mutation (L885R) in the guanylyl cyclase domain of the *Npr2* gene that prevents the protein from catalyzing cGMP formation [39] (Jackson Laboratories, Stock Number 003913). Animals were maintained on a mixed genetic background. Mice were genotyped using previously described PCR protocols (Jackson Laboratories, [33], [39]). For timed pregnancies, embryonic day 0.5 (E0.5) was defined as noon on the day of a copulatory plug. In most cases, animals were euthanized using C0_2_ exposure followed by cervical dislocation or anesthetized with ketamine/xylazine and then either cervically dislocated or perfused transcardially with fixative. For physiological studies, young animals were decapitated with colostomy scissors. All mice were maintained in accordance with institutional and National Institutes of Health (NIH) guidelines approved by the Institutional Animal Care and Use Committees (IACUC) at Harvard Medical School (Protocol 03611) and University of Wisconsin (Protocol M00449-0-12-12).

### Tonotopic dye labeling

E16.5 embryo heads were fixed in 4% paraformaldehyde (PFA) in PBS overnight and rinsed in PBS. The cochlea was exposed so that basal and apical turns were visible. In some cases, a small crystal of DiI (Life Technologies) was placed in the base of the cochlea, while a crystal of DiD (Life Technologies) was placed in the apex. In other cases, a picospritzer was used to inject a small amount of DiI or DiD dissolved in DMSO into the base or apex of the cochlea, respectively. Tissue was incubated at 37°C in PBS for 3–4 days to allow the dye to diffuse along axons. The hindbrain was then dissected out, cleared in ScaleA2 [66] at 37°C for 1 hour, mounted on a slide, and imaged by confocal microscopy to obtain z-projection images. To determine caudal/rostral bias of projections, the bifurcation zone was demarcated with a 100 pixel (px) diameter circle, and the intensity of caudal and rostral projections was measured by defining 100 px diameter circles adjacent to this zone. The ratio of caudal to rostral projections was calculated for each image and averaged for controls (n = 2 wild-type+n = 2 heterozygote embryos) and *Npr2* mutants (n = 4 embryos). Student's t-test was used to assess statistical significance.

For P14–P18 animals, mice were perfused transcardially with 4% PFA in PBS (n = 2 control and 4 *Npr2* mutants). Their heads were bisected sagittally and fixed overnight in 4% PFA in PBS at 4°C. Tissue was rinsed in PBS and dissected so that the cochlea was exposed, with the brain still attached, then decalcified in 0.1 M EDTA in PBS at room temperature for 3 days. The decalcified bone covering the organ of Corti was removed so that mid and apical turns were visible, and small crystals of DiI and DiD were placed in the apical and mid-turns of the cochlea, respectively, using a 30-gauge needle to first create a small slit into which the dye crystal could be inserted. The tissue was incubated at 37°C in 4% PFA in PBS for 1 week, at which point most of the dye had diffused along projections. Since the axons are heavily myelinated at this stage and require a large amount of dye to reach the central projections in the cochlear nucleus, an additional crystal of DiI or DiD was at this time placed in the same slit, and allowed to diffuse for another week. The cochlea and cochlear nuclei were then dissected out, embedded in 5% low melt agarose in PBS, and sectioned by vibratome at 150 µm. For the cochlea, transverse sections of the cochlear nerve were collected, and for the cochlear nucleus, transverse sections of the aVCN and pVCN were collected. These were mounted on a slide and imaged by confocal microscopy (Leica SP8 X).

### Visualization of cochlear afferents

To examine the overall pattern of peripheral projections in the cochlea, wild-type (n = 2) or *Npr2* mutant (n = 2) animals were perfused transcardially with 4% PFA in PBS, and the cochleae were dissected out and fixed overnight at 4°C, then subjected to whole-mount immunofluorescence with chick anti-Neurofilament antibody (1∶1000, Abcam), using Alexa488-conjugated goat anti-chick secondary antibody (1∶1000, Life Technologies). Cochleae were mounted in Vectashield (Vector Labs) and imaged on a Leica SP8 X confocal microscope. To visualize individual SGN peripheral processes in the cochlea, *Npr2^cn/+^* mice were crossed with *Npr2* heterozygotes also carrying the *Neurog1-creER^T2^* and *Ai14: tdTomato* alleles. Since leaky Cre expression results in random, sparse recombination of the *Ai14: tdTomato* allele even without tamoxifen administration, this allowed us to label relatively few SGNs with the red fluorescent protein tdTomato. Cochleae from perfusion-fixed P14 animals (n = 3 heterozygous control, n = 5 *Npr2* mutant) were collected and further fixed overnight in 4% PFA in PBS, then mounted on a slide in Vectashield for confocal microscopy (Leica SP8 X).

To label SGN central axons at E16.5, embryo heads were fixed in 4% PFA in PBS overnight and the cochlea was exposed. A picospritzer was used to inject DiI dissolved in DMSO into the cochlea, and then treated as described above for tonotopic dye labeling. To visualize the overall pattern of SGN projections in the cochlear nuclei at postnatal stages, *Npr2^cn/+^* mice were mated with *Npr2* heterozygotes also carrying the *Neurog1-creER^T2^* and *Ai14: tdTomato* alleles. P14–P18 animals (n = 5 heterozygous control, n = 6 *Npr2* mutant) were perfused transcardially with 4% PFA in PBS, and their brains were drop fixed overnight in 4% PFA in PBS. Cochlear nuclei were then dissected out and cleared overnight in ScaleA2. The entire cochlear nucleus was mounted in ScaleA2 on a glass slide and imaged using a Leica SP8 X confocal microscope. Tiled confocal stacks (∼300 µm thick) were obtained at 10× so that the entire cochlear nucleus was covered. These tiled images were stitched together by ImageJ and z-projected to generate a single, large image of the cochlear nucleus including aVCN, pVCN, and DCN. For examination of projections in just the aVCN and pVCN, animals were processed as above, and then cochlear nuclei were embedded in 5% low melt agarose in PBS and cut sagittally at 150 µm using a vibratome. Regular confocal stacks were obtained at 20× and 40× and z-projected.

### Biocytin injections

To assess the morphology of auditory nerve fibers and topographic organization of tuberculoventral cell projections, biocytin injections were made into the aVCN in parasagittal slices in mice aged between P14 and P26. With a single, parasagittal cut, the cochlear nuclei were removed from the brainstem in a single “slice” of up to 400 µm either with a vibratome or with scissors. The slice was maintained *in vitro* as in electrophysiological experiments. With a picospritzer, normal saline containing 1% biocytin (Sigma) was injected into the aVCN through a pipette with a tip diameter of ∼5 µm. Movement of the pipette through the slice disrupted processes that crossed the injection site as pulses of pressure released biocytin. Biocytin was allowed to spread through the tissue for 1.5 to 2 hours as slices continued to be superfused with warmed, oxygenated saline. Slices were then fixed in 4% PFA, stored at 4°C, embedded in a gelatin-albumin mixture, and resectioned at 40 to 60 µm in frozen sections. Biocytin in cells and fibers was visualized with horseradish peroxidase (Vectastain ABC Elite Kit, Vector Laboratories) [12]. Photomicrographs were taken through a Zeiss Axioskop with a Zeiss Axiocam.

### Quantification of TV cell tonotopic mapping

After being processed histologically, sections were analyzed with a *camera lucida*. Each section was reconstructed and marked with the locations of labeled neurons and landmarks. Landmarks were used to reconstruct slices as illustrated in Figure 5D. The distribution of labeled cells in the reconstructed slice was measured by means of a transparent grid that was laid parallel to an isofrequency band. Cells were then counted within parallel rows of squares as illustrated by the histograms in Figure 5D. Comparisons between genotypes were made by lining up peaks in histograms and summing cells in bands. Half widths were statistically compared using a one-way ANOVA test with Origin (v 7.5) software.

### Electron microscopy

P21 animals were perfused transcardially with 4% PFA in PBS, and bisected heads were fixed overnight at 4°C in fixative (2.5% PFA, 5% glutaraldehyde, 0.06% picric acid in 0.2 M sodium cacodylate buffer). The cochlear nuclei were dissected out with the eighth nerve attached, and the region where the eighth nerve enters the cochlear nuclei was cut into a 1–2 mm cube in the fixative. The tissue was washed in 0.2 M sodium cadocylate buffer three times, followed by incubation in 1% osmium tetroxide/1.5% potassium ferrocyanide in water for 1 hour in the dark at room temperature. After three washes in malelate buffer (pH 5.15), the tissue was placed in 1% Uranyl Acetate or maleate buffer for 30 minutes, washed in water three times, and then dehydrated through an ethanol series (70% ethanol for 15 min, 90% ethanol for 15 min, and 100% ethanol twice for 15 min). Tissue was incubated in propyleneoxide solution for 1 hour, and then infiltrated with Epon resin mixed 1∶1 with propyleneoxide for 2–3 hours at room temperature. Samples were embedded in freshly mixed Epon and polymerized for 24–48 hours at 60°C. Thin sections were cut transverse to the eighth nerve using a Reichert Ultracut-S and were imaged using a Technai G2 Spirit BioTWIN transmission electron microscope with an AMT 2k CCD camera. To calculate the g-ratio, EM images of the eighth nerve were obtained for control (n = 3) and *Npr2* mutant (n = 3) mice, and Fiji (ImageJ) was used to demarcate the area encompassed by each axon, as well as the area of the entire myelinated fiber. The g-ratio for each axon was calculated for ∼200 axons for each animal by dividing the diameter of the entire myelinated fiber by the diameter of the axon proper, and averages for controls and mutants were calculated. Statistical significance was assessed using Student's t-test.

### Auditory brainstem recordings

Auditory brainstem responses (ABRs) were recorded in 6-week-old mice in a soundproof chamber, as previously described [67]. Average ABR waveforms were plotted using a MATLAB (MathWorks) script written by Ann E. Hickox in the laboratory of Dr. Charles Liberman (Eaton Peabody Laboratories, Massachusetts Eye and Ear Infirmary, Boston, MA). Statistical significance was assessed using Student's t-test.

### Preparation of slices

Coronal slices of the cochlear nuclei were made from mice between P17 and P25. Slices (220 µm thick) were cut with a vibrating microtome (Leica VT 1000S) in normal physiological saline or in saline with reduced Na^+^ at 24–27°C, and then transferred to a recording chamber (∼0.6 ml) and superfused continually at 5–6 ml/min. Temperature was controlled with a Thermalert thermometer (Physitemp) the input of which comes from a small thermistor (IT-23, Physitemp, diameter: 0.1 mm) placed between the inflow of the chamber and the tissue. The output of the Thermalert thermometer was fed into a custom-made, feedback-controlled heater that heated the saline in glass tubing (1.5 mm) just before it reached the chamber to maintain the temperature at 33°C. Biocytin injections were made under the control of a Wild (M5) dissecting microscope. For electrophysiological recordings, the tissue was visualized through a compound microscope (Zeiss Axioskop) with a 63× water immersion objective and CCD Camera (Hamamatsu), with the image displayed on a video screen.

### Electrophysiological recordings

Whole-cell patch clamp recordings were made by using an Axopatch 200A amplifier (Axon Instruments, Burlingame, CA). Patch electrodes whose resistances ranged between 3.5 and 8 MΩ were made from borosilicate glass. All recordings of eEPSCs were digitized at 40 kHz and low-pass filtered at 10 kHz. The series resistance was compensated by 85–90% in recordings from octopus cells and by 70–80% in recordings from T stellate and bushy cells with a 10-µsec lag [16]. EPSCs were evoked by shocks through a Master-8 stimulator and Iso-flex isolator (AMPI, Jerusalem, Israel), delivered through an extracellular-saline-filled glass pipette (∼5 µm tip). Analysis of EPSCs was performed by using pClamp (Clampfit 9.0, Axon Instruments).

For solutions, all chemicals were from Sigma-Aldrich, unless stated otherwise.

*Normal saline:* The normal extracellular physiological saline comprised (in mM) 130 NaCl, 3 KCl, 1.2 KH_2_PO_4_, 2.4 CaCl_2_, 1.3 MgSO_4_, 20 NaHCO_3_, 6 HEPES, 10 glucose, and 0.4 ascorbic acid saturated with 95% O_2_-5% CO_2_, pH 7.3–7.4, between 24 and 33°C. The osmolality was 306 mOsm/kg (3D3 Osmometer, Advanced Instruments Inc, Norwood, MA). *Cutting solution:* Some dissections were performed in a special cutting solution that contained (in mM) 99 NaCl, 3 KCl, 1.2 KH_2_PO_4_, 1 CaCl_2_, 1.3 MgSO_4_, 20 NaHCO_3_, 6 HEPES, 10 glucose, and 72 sucrose.

*Pipette solution:* Recording pipettes were filled with a solution that consisted of (in mM) 90 Cs_2_SO_4_, 20 CsCl, 5 EGTA, 10 HEPES, 4 Mg-ATP, 0.3 GTP, 5 Na-phosphocreatine, 5 mM QX314, and was adjusted to pH 7.3 with CsOH (∼298 mOsm). Voltages were corrected for a −10 mV junction potential.

## Supporting Information

Figure S1 Responses to depolarizing and hyperpolarizing current pulses reveal the intrinsic electrical properties of neurons. The traces show responses to the three major classes of principal cells of the ventral cochlear nucleus: bushy (top), T stellate (middle) and octopus cells (bottom), from wild type (left) and mutant (right) mice. Each family of traces shows superimposed responses to the injection of depolarizing and hyperpolarizing current pulses. Bushy and T stellate cell responses were to current pulses between −0.5 and +0.2 nA in 0.05 nA steps; octopus cell responses were to current pulses between −3.5 and +3 nA in 0.5 nA steps. Please note that the voltage scales are different between cell types but consistent within cell types. The responses to current are characteristic of the principal cells making it possible to distinguish cell types electrophysiologically. The responses are similar in cells from wild type and mutant mice.
(PDF)
