# Supplementary figures and images for: Mutation of Npr2 Leads to Blurred Tonotopic Organization of Central Auditory Circuits in Mice
Source: PLoS Genet. 2014 Dec 4;10(12):e1004823. doi: 10.1371/journal.pgen.1004823 (PMC4256264; doi:10.1371/journal.pgen.1004823)

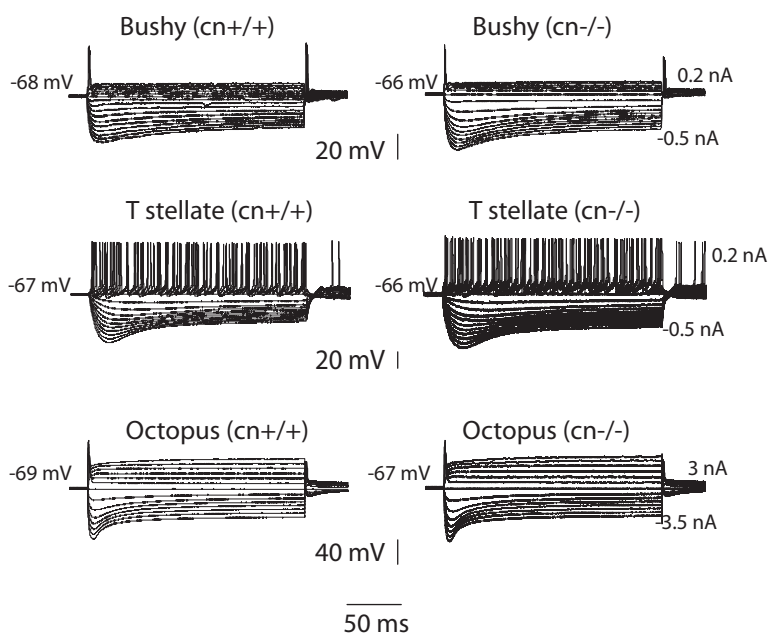

Supplement: Figure S1 — Responses to depolarizing and hyperpolarizing current pulses reveal the intrinsic electrical properties of neurons. The traces show responses to the three major classes of principal cells of the ventral cochlear nucleus: bushy (top), T stellate (middle) and octopus cells (bottom), from wild type (left) and mutant (right) mice. Each family of traces shows superimposed responses to the injection of depolarizing and hyperpolarizing current pulses. Bushy and T stellate cell responses were to current pulses between −0.5 and +0.2 nA in 0.05 nA steps; octopus cell responses were to current pulses between −3.5 and +3 nA in 0.5 nA steps. Please note that the voltage scales are different between cell types but consistent within cell types. The responses to current are characteristic of the principal cells making it possible to distinguish cell types electrophysiologically. The responses are similar in cells from wild type and mutant mice. (PDF) [file pgen.1004823.s001.pdf]
